# (*E*)-1-(2,4-Di­nitro­phen­yl)-2-(3-eth­oxy-4-hy­droxy­benzyl­idene)hydrazine

**DOI:** 10.1107/S1600536813033989

**Published:** 2013-12-24

**Authors:** Hoong-Kun Fun, Suchada Chantrapromma, Pumsak Ruanwas, Thawanrat Kobkeatthawin, C. S. Chidan Kumar

**Affiliations:** a X-ray Crystallography Unit, School of Physics, Universiti Sains Malaysia, 11800 USM, Penang, Malaysia; b Department of Chemistry, Faculty of Science, Prince of Songkla University, Hat-Yai, Songkhla 90112, Thailand

## Abstract

The mol­ecule of the title hydrazine derivative, C_15_H_14_N_4_O_6_, is essentially planar, the dihedral angle between the substituted benzene rings being 2.25 (9)°. The eth­oxy and hy­droxy groups are almost coplanar with their bound benzene ring [r.m.s. deviation = 0.0153 (2) Å for the ten non-H atoms]. Intra­molecular N—H⋯O and O—H⋯O_eth­oxy_ hydrogen bonds generate *S*(6) and *S*(5) ring motifs, respectively. In the crystal, mol­ecules are linked by O—H⋯O_nitro_ hydrogen bonds into chains propagating in [010]. Weak aromatic π–π inter­actions, with centroid–centroid distances of 3.8192 (19) and 4.0491 (19) Å, are also observed.

## Related literature   

For a related structure and background to hydrazones, see: Fun *et al.* (2013 ▶). For other related structures, see: Fun *et al.* (2011 ▶, 2012 ▶). For the measurement of anti-oxidant activity, see: Molyneux (2004 ▶).
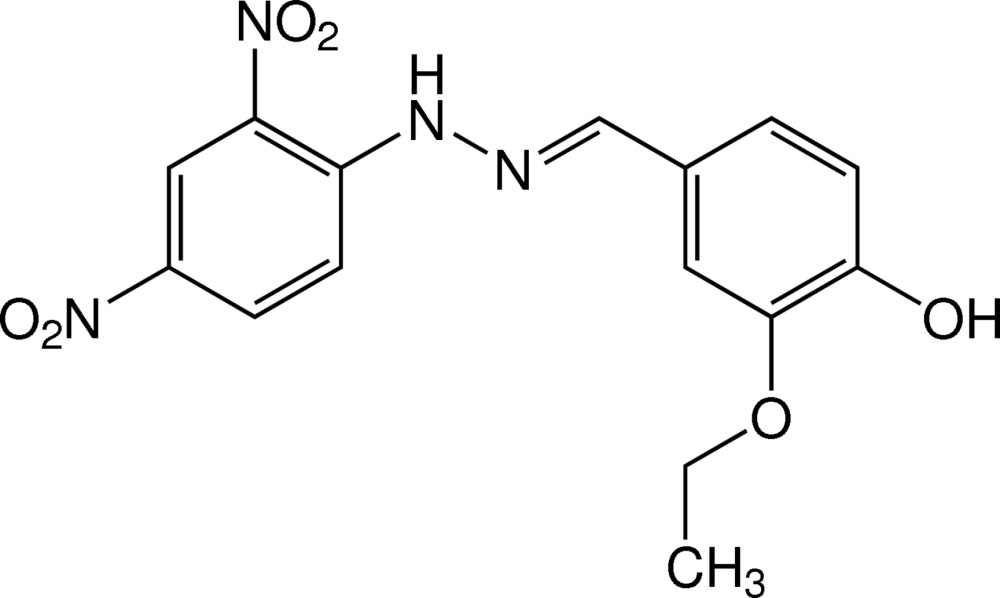


## Experimental   

### 

#### Crystal data  

C_15_H_14_N_4_O_6_
*M* *_r_* = 346.30
Monoclinic, 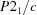

*a* = 10.245 (4) Å
*b* = 13.679 (5) Å
*c* = 14.184 (5) Å
β = 129.15 (2)°
*V* = 1541.5 (11) Å^3^
*Z* = 4
Mo *K*α radiation
μ = 0.12 mm^−1^
*T* = 298 K
0.52 × 0.37 × 0.07 mm

#### Data collection  

Bruker APEXII CCD diffractometer
Absorption correction: multi-scan (*SADABS*; Bruker, 2009 ▶) *T*_min_ = 0.941, *T*_max_ = 0.992
16113 measured reflections
4060 independent reflections
2183 reflections with *I* > 2σ(*I*)
*R*_int_ = 0.036

#### Refinement  

*R*[*F* ^2^ > 2σ(*F* ^2^)] = 0.047
*wR*(*F* ^2^) = 0.131
*S* = 1.01
4060 reflections
235 parameters
H atoms treated by a mixture of independent and constrained refinement
Δρ_max_ = 0.16 e Å^−3^
Δρ_min_ = −0.22 e Å^−3^

### 

Data collection: *APEX2* (Bruker, 2009 ▶); cell refinement: *SAINT* (Bruker, 2009 ▶); data reduction: *SAINT*; program(s) used to solve structure: *SHELXTL* (Sheldrick, 2008 ▶); program(s) used to refine structure: *SHELXTL*; molecular graphics: *SHELXTL*; software used to prepare material for publication: *SHELXTL*, *PLATON* (Spek, 2009 ▶), *Mercury* (Macrae *et al.*, 2006 ▶) and *publCIF* (Westrip, 2010 ▶).

## Supplementary Material

Crystal structure: contains datablock(s) global, I. DOI: 10.1107/S1600536813033989/hb7175sup1.cif

Structure factors: contains datablock(s) I. DOI: 10.1107/S1600536813033989/hb7175Isup2.hkl

Supporting information file. DOI: 10.1107/S1600536813033989/hb7175Isup3.cml

Additional supporting information:  crystallographic information; 3D view; checkCIF report

## Figures and Tables

**Table 1:** Hydrogen-bond geometry (Å, °)

*D*—H⋯*A*	*D*—H	H⋯*A*	*D*⋯*A*	*D*—H⋯*A*
O6—H1*O*6⋯O2^i^	0.84 (3)	2.21 (3)	2.986 (2)	155 (3)
O6—H1*O*6⋯O5	0.84 (3)	2.19 (3)	2.663 (3)	116 (2)
N1—H1*N*1⋯O1	0.86 (2)	2.007 (18)	2.641 (2)	130.0 (18)

Symmetry code: (i) 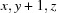.

**Fig. 1.**
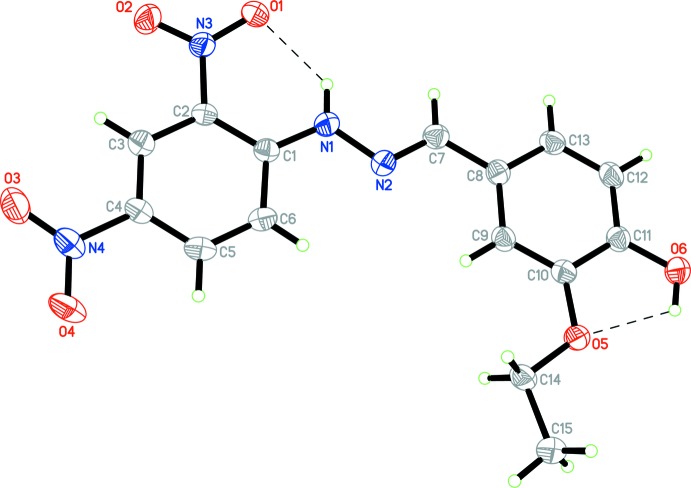
The molecular structure of (I), showing 40% probability displacement ellipsoids. Intramolecular N—H···O and O—H···O hydrogen bonds are shown as dashed lines.

**Fig. 2.**
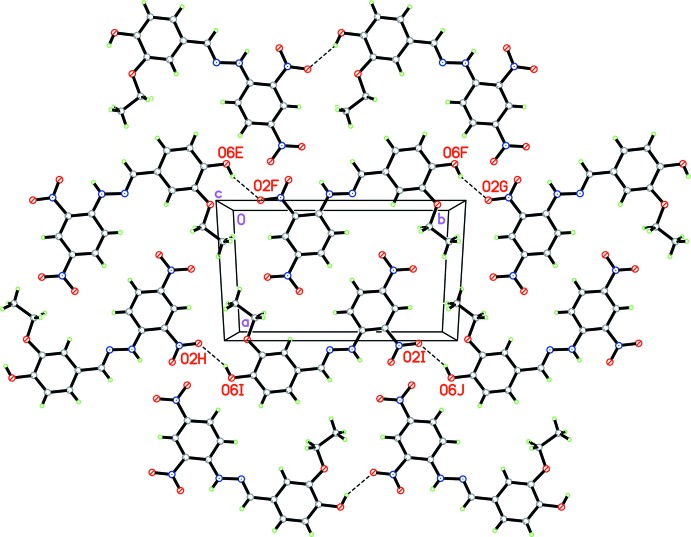
The crystal packing of (I) viewed along the c axis. Hydrogen bonds are shown as dashed lines.

**Fig. 3.**
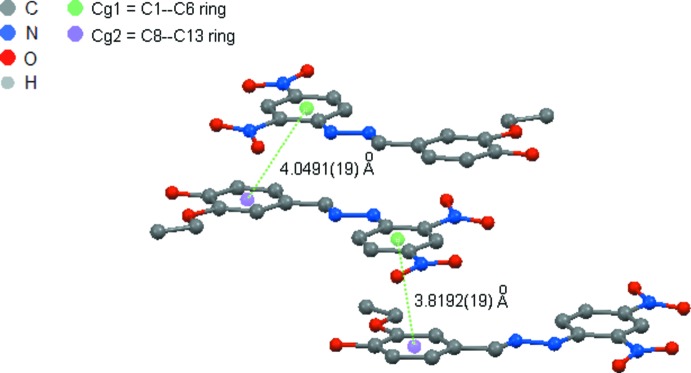
π-π interactions between aromatic rings. H-atoms are omitted for clarify.

## supplementary crystallographic information

### 1. Comment

As part of our on-going research on diaryl-hydrazones with potential bioactivity, the title compound (I) was synthesized in order to study and compare its antioxidant activity with the other related compounds (Fun *et al.*, 2011; 2012; 2013). In our antioxidant activity evaluation of (I) by DPPH scavenging (Molyneux, 2004) it was found that (I) possesses strong antioxidant activity with 82.71% inhibition, comparison with L-ascorbic acid as a standard (90.39 % inhibition). Herein we report the synthesis and crystal structure of (I).

In Fig. 1, the molecular structure of (I), C_15_H_14_N_4_O_6_, is essentially planar with the dihedral angle between the two substituted benzene rings being 2.25 (9)°. The mean plane through the bridge fragment (N1/N2/C7) makes the dihedral angles of 2.25 (19) and 2.30 (19)° with the C1–C6 and C8–C13 rings, respectively. Both nitro groups of the 2,4-dinitrophenyl are slightly deviated with respect to their attached ring [torsion angles O1—N3—C2—C1 = -8.3 (2)°, O2—N3—C2—C3 = -9.4 (2)°, O3—N4—C4—C3 = -7.0 (2)° and O4—N4—C4—C5 = -7.2 (2)°]. The substituted ethoxy and hydroxy groups are co-planar with the bound benzene ring with the *r.m.s*. deviation of 0.0153 (2) Å for the ten non H atoms and the torsion angles C9—C10—O5—C14 = -3.1 (2)° and C15—C14—O5—C10 = -178.05 (14)°. Intramolecular N1—H1N1···O1 hydrogen bond (Fig. 1 and Table 1) generates an S(6) ring motif whereas another intramolecular O6—H1O6—O5 hydrogen bond generates S(5) ring motif. These intramolecular hydrogen bonds help to stabilize the planarity of the molecule. Bond distances in (I) are comparable with those observed in the closely related structure (Fun *et al.*, 2013).

In the crystal (Fig. 2), the molecules are linked by intermolecular O6—H1O6···O2 hydrogen bond (Table 1) into chains along [010]. There are weak π–π interactions (Fig. 3) with the distances of Cg_1_···Cg_2_^ii^ = 3.8192 (19) Å and Cg_1_···Cg_2_^iii^ = 4.0491 (19) Å [symmetry codes (ii) = 2-x, -1/2+y, 1/2-z (iii) 2-x, -y, -z]; Cg_1_ and Cg_2_ are the centroids of C1–C6 and C8–C13 rings, respectively.

### 2. Experimental

2,4-dinitrophenylhydrazine (0.40 g, 2 mmol) was dissolved in ethanol (10 ml) and H_2_SO_4_ (conc.) (98 %, 0.50 ml) was added slowly with stirring. A solution of 3-ethoxy-4-hydroxybenzaldehyde (0.30 g, 2 mmol) in ethanol (20 ml) was then added to the solution with continuous stirring for 1 hr, yielding an orange solid which was filtered off and washed with methanol. Orange plates of (I) were recrystallized from acetone solution by slow evaporation of the solvent at room temperature over a few weeks, Mp. 515-516 K.

### 3. Refinement

Hydrazine and hydroxy H atoms were located from a difference Fourier map and refined freely. The remaining H atoms were positioned geometrically and allowed to ride on their parent atoms, with d(C-H) = 0.93 Å for CH and aromatic, and 0.96 Å for CH_3_ atoms. The *U*_iso_ values were constrained to be 1.5*U*_eq_ of the carrier atom for methyl H atoms and 1.2*U*_eq_ for the remaining H atoms. A rotating group model was used for the methyl groups.

### Crystal data

**Table:**

C_15_H_14_N_4_O_6_	*F*(000) = 720
*M**_r_* = 346.30	*D*_x_ = 1.492 Mg m^−^^3^
Monoclinic, *P*2_1_/*c*	Melting point = 515–516 K
Hall symbol: -P 2ybc	Mo *K*α radiation, λ = 0.71073 Å
*a* = 10.245 (4) Å	Cell parameters from 4060 reflections
*b* = 13.679 (5) Å	θ = 2.4–29.0°
*c* = 14.184 (5) Å	µ = 0.12 mm^−^^1^
β = 129.15 (2)°	*T* = 298 K
*V* = 1541.5 (11) Å^3^	Plate, orange
*Z* = 4	0.52 × 0.37 × 0.07 mm

### Data collection

**Table:**

Bruker APEXII CCD diffractometer	4060 independent reflections
Radiation source: sealed tube	2183 reflections with *I* > 2σ(*I*)
Graphite monochromator	*R*_int_ = 0.036
φ and ω scans	θ_max_ = 29.0°, θ_min_ = 2.4°
Absorption correction: multi-scan (*SADABS*; Bruker, 2009)	*h* = −13→13
*T*_min_ = 0.941, *T*_max_ = 0.992	*k* = −18→18
16113 measured reflections	*l* = −13→19

### Refinement

**Table:**

Refinement on *F*^2^	Primary atom site location: structure-invariant direct methods
Least-squares matrix: full	Secondary atom site location: difference Fourier map
*R*[*F*^2^ > 2σ(*F*^2^)] = 0.047	Hydrogen site location: inferred from neighbouring sites
*wR*(*F*^2^) = 0.131	H atoms treated by a mixture of independent and constrained refinement
*S* = 1.01	*w* = 1/[σ^2^(*F*_o_^2^) + (0.0566*P*)^2^ + 0.0702*P*] where *P* = (*F*_o_^2^ + 2*F*_c_^2^)/3
4060 reflections	(Δ/σ)_max_ = 0.001
235 parameters	Δρ_max_ = 0.16 e Å^−^^3^
0 restraints	Δρ_min_ = −0.22 e Å^−^^3^

### Special details

**Table:**

Geometry. All esds (except the esd in the dihedral angle between two l.s. planes) are estimated using the full covariance matrix. The cell esds are taken into account individually in the estimation of esds in distances, angles and torsion angles; correlations between esds in cell parameters are only used when they are defined by crystal symmetry. An approximate (isotropic) treatment of cell esds is used for estimating esds involving l.s. planes.
Refinement. Refinement of F^2^ against ALL reflections. The weighted R-factor wR and goodness of fit S are based on F^2^, conventional R-factors R are based on F, with F set to zero for negative F^2^. The threshold expression of F^2^ > 2sigma(F^2^) is used only for calculating R-factors(gt) etc. and is not relevant to the choice of reflections for refinement. R-factors based on F^2^ are statistically about twice as large as those based on F, and R- factors based on ALL data will be even larger.

### Fractional atomic coordinates and isotropic or equivalent isotropic displacement parameters (Å^2^)

**Table:**

	*x*	*y*	*z*	*U*_iso_*/*U*_eq_	
O1	1.20079 (13)	−0.22891 (8)	0.16025 (12)	0.0622 (4)	
O2	1.05019 (14)	−0.35272 (9)	0.13142 (13)	0.0736 (5)	
O3	0.46877 (15)	−0.32518 (12)	−0.07366 (14)	0.0814 (5)	
O4	0.36011 (15)	−0.18505 (11)	−0.08629 (13)	0.0767 (4)	
O5	1.01832 (12)	0.42468 (8)	0.13492 (11)	0.0532 (3)	
O6	1.32309 (16)	0.49799 (9)	0.24070 (13)	0.0580 (4)	
H1O6	1.230 (3)	0.5246 (19)	0.209 (2)	0.121 (10)*	
N1	1.09605 (18)	−0.05017 (10)	0.15152 (14)	0.0489 (4)	
H1N1	1.183 (2)	−0.0854 (13)	0.1800 (17)	0.065 (6)*	
N2	1.10133 (17)	0.05030 (9)	0.15757 (13)	0.0472 (4)	
N3	1.06865 (16)	−0.26439 (10)	0.13050 (13)	0.0494 (4)	
N4	0.47883 (17)	−0.23628 (13)	−0.05806 (14)	0.0594 (4)	
C1	0.94828 (19)	−0.09698 (11)	0.10069 (14)	0.0411 (4)	
C2	0.93039 (18)	−0.19991 (11)	0.09135 (14)	0.0409 (4)	
C3	0.77777 (19)	−0.24509 (12)	0.04025 (14)	0.0452 (4)	
H3A	0.7692	−0.3129	0.0357	0.054*	
C4	0.63954 (19)	−0.18842 (12)	−0.00355 (15)	0.0463 (4)	
C5	0.6511 (2)	−0.08732 (13)	0.00279 (16)	0.0523 (5)	
H5A	0.5558	−0.0499	−0.0284	0.063*	
C6	0.8015 (2)	−0.04225 (12)	0.05462 (16)	0.0509 (4)	
H6A	0.8077	0.0256	0.0598	0.061*	
C7	1.2451 (2)	0.08997 (12)	0.20969 (15)	0.0474 (4)	
H7A	1.3388	0.0507	0.2419	0.057*	
C8	1.26408 (19)	0.19596 (11)	0.21919 (14)	0.0432 (4)	
C9	1.12420 (19)	0.25734 (11)	0.17161 (15)	0.0437 (4)	
H9A	1.0192	0.2301	0.1360	0.052*	
C10	1.14273 (18)	0.35742 (11)	0.17773 (15)	0.0432 (4)	
C11	1.30253 (19)	0.39867 (12)	0.23335 (15)	0.0448 (4)	
C12	1.43888 (19)	0.33878 (12)	0.28036 (16)	0.0510 (5)	
H12A	1.5442	0.3661	0.3172	0.061*	
C13	1.42040 (19)	0.23804 (12)	0.27326 (15)	0.0485 (4)	
H13A	1.5134	0.1982	0.3049	0.058*	
C14	0.84992 (18)	0.38972 (12)	0.07237 (16)	0.0509 (4)	
H14A	0.8445	0.3497	0.1263	0.061*	
H14B	0.8145	0.3504	0.0029	0.061*	
C15	0.7378 (2)	0.47736 (13)	0.03131 (17)	0.0573 (5)	
H15A	0.6249	0.4563	−0.0071	0.086*	
H15B	0.7392	0.5143	−0.0256	0.086*	
H15C	0.7775	0.5175	0.1003	0.086*	

### Atomic displacement parameters (Å^2^)

**Table:**

	*U*^11^	*U*^22^	*U*^33^	*U*^12^	*U*^13^	*U*^23^
O1	0.0395 (7)	0.0560 (8)	0.0861 (10)	0.0018 (5)	0.0373 (7)	−0.0034 (7)
O2	0.0515 (8)	0.0417 (8)	0.1069 (12)	0.0051 (6)	0.0401 (8)	0.0016 (7)
O3	0.0537 (8)	0.0706 (10)	0.1065 (12)	−0.0078 (7)	0.0442 (9)	0.0088 (9)
O4	0.0417 (7)	0.0993 (11)	0.0876 (11)	0.0102 (7)	0.0400 (8)	0.0084 (8)
O5	0.0389 (6)	0.0431 (6)	0.0697 (8)	−0.0035 (5)	0.0305 (6)	−0.0059 (6)
O6	0.0490 (7)	0.0453 (7)	0.0754 (9)	−0.0079 (6)	0.0373 (7)	−0.0069 (6)
N1	0.0437 (8)	0.0400 (8)	0.0605 (10)	0.0040 (6)	0.0317 (8)	0.0026 (7)
N2	0.0511 (8)	0.0400 (8)	0.0540 (9)	0.0007 (6)	0.0349 (8)	0.0014 (6)
N3	0.0402 (8)	0.0443 (9)	0.0555 (9)	0.0026 (6)	0.0264 (7)	−0.0023 (7)
N4	0.0415 (8)	0.0740 (11)	0.0609 (10)	0.0004 (8)	0.0314 (8)	0.0091 (9)
C1	0.0416 (9)	0.0437 (9)	0.0409 (9)	0.0031 (7)	0.0273 (8)	0.0016 (7)
C2	0.0359 (8)	0.0435 (9)	0.0430 (9)	0.0053 (7)	0.0248 (8)	0.0021 (7)
C3	0.0439 (9)	0.0476 (9)	0.0433 (10)	0.0018 (7)	0.0272 (8)	0.0024 (8)
C4	0.0373 (8)	0.0572 (11)	0.0457 (10)	0.0029 (7)	0.0269 (8)	0.0042 (8)
C5	0.0443 (10)	0.0602 (12)	0.0538 (11)	0.0141 (8)	0.0316 (9)	0.0064 (9)
C6	0.0512 (10)	0.0449 (10)	0.0597 (12)	0.0094 (8)	0.0364 (10)	0.0022 (8)
C7	0.0459 (9)	0.0479 (10)	0.0477 (10)	0.0039 (7)	0.0292 (9)	0.0029 (8)
C8	0.0438 (9)	0.0453 (10)	0.0411 (9)	−0.0005 (7)	0.0271 (8)	0.0018 (7)
C9	0.0369 (8)	0.0462 (10)	0.0449 (10)	−0.0053 (7)	0.0243 (8)	−0.0035 (8)
C10	0.0388 (8)	0.0470 (10)	0.0432 (10)	0.0001 (7)	0.0257 (8)	−0.0017 (8)
C11	0.0434 (9)	0.0459 (10)	0.0454 (10)	−0.0060 (7)	0.0282 (8)	−0.0048 (8)
C12	0.0386 (9)	0.0563 (11)	0.0564 (12)	−0.0071 (8)	0.0292 (9)	−0.0023 (9)
C13	0.0378 (9)	0.0538 (10)	0.0511 (11)	0.0038 (7)	0.0268 (8)	0.0042 (8)
C14	0.0371 (9)	0.0534 (10)	0.0586 (11)	−0.0046 (7)	0.0285 (9)	−0.0071 (9)
C15	0.0494 (10)	0.0560 (11)	0.0656 (13)	0.0055 (8)	0.0359 (10)	0.0026 (9)

### Geometric parameters (Å, º)

**Table:**

O1—N3	1.2349 (16)	C5—C6	1.366 (2)
O2—N3	1.2243 (17)	C5—H5A	0.9300
O3—N4	1.229 (2)	C6—H6A	0.9300
O4—N4	1.2312 (18)	C7—C8	1.458 (2)
O5—C10	1.3647 (18)	C7—H7A	0.9300
O5—C14	1.4351 (18)	C8—C13	1.390 (2)
O6—C11	1.369 (2)	C8—C9	1.412 (2)
O6—H1O6	0.84 (3)	C9—C10	1.377 (2)
N1—C1	1.358 (2)	C9—H9A	0.9300
N1—N2	1.3759 (18)	C10—C11	1.411 (2)
N1—H1N1	0.858 (17)	C11—C12	1.375 (2)
N2—C7	1.279 (2)	C12—C13	1.386 (2)
N3—C2	1.4490 (19)	C12—H12A	0.9300
N4—C4	1.459 (2)	C13—H13A	0.9300
C1—C2	1.415 (2)	C14—C15	1.500 (2)
C1—C6	1.417 (2)	C14—H14A	0.9700
C2—C3	1.386 (2)	C14—H14B	0.9700
C3—C4	1.372 (2)	C15—H15A	0.9600
C3—H3A	0.9300	C15—H15B	0.9600
C4—C5	1.386 (2)	C15—H15C	0.9600
			
C10—O5—C14	118.10 (12)	C8—C7—H7A	119.6
C11—O6—H1O6	108.7 (18)	C13—C8—C9	119.05 (15)
C1—N1—N2	119.40 (13)	C13—C8—C7	120.17 (15)
C1—N1—H1N1	117.7 (12)	C9—C8—C7	120.77 (14)
N2—N1—H1N1	122.9 (12)	C10—C9—C8	120.21 (14)
C7—N2—N1	116.45 (14)	C10—C9—H9A	119.9
O2—N3—O1	121.84 (13)	C8—C9—H9A	119.9
O2—N3—C2	118.99 (13)	O5—C10—C9	126.10 (14)
O1—N3—C2	119.17 (14)	O5—C10—C11	114.04 (14)
O3—N4—O4	123.46 (15)	C9—C10—C11	119.86 (14)
O3—N4—C4	118.54 (14)	O6—C11—C12	119.61 (14)
O4—N4—C4	118.00 (17)	O6—C11—C10	120.53 (14)
N1—C1—C2	123.64 (14)	C12—C11—C10	119.86 (15)
N1—C1—C6	119.91 (15)	C11—C12—C13	120.44 (15)
C2—C1—C6	116.45 (14)	C11—C12—H12A	119.8
C3—C2—C1	121.96 (14)	C13—C12—H12A	119.8
C3—C2—N3	115.85 (14)	C12—C13—C8	120.57 (15)
C1—C2—N3	122.15 (13)	C12—C13—H13A	119.7
C4—C3—C2	119.08 (16)	C8—C13—H13A	119.7
C4—C3—H3A	120.5	O5—C14—C15	107.50 (14)
C2—C3—H3A	120.5	O5—C14—H14A	110.2
C3—C4—C5	120.88 (15)	C15—C14—H14A	110.2
C3—C4—N4	118.90 (16)	O5—C14—H14B	110.2
C5—C4—N4	120.22 (14)	C15—C14—H14B	110.2
C6—C5—C4	120.41 (15)	H14A—C14—H14B	108.5
C6—C5—H5A	119.8	C14—C15—H15A	109.5
C4—C5—H5A	119.8	C14—C15—H15B	109.5
C5—C6—C1	121.21 (16)	H15A—C15—H15B	109.5
C5—C6—H6A	119.4	C14—C15—H15C	109.5
C1—C6—H6A	119.4	H15A—C15—H15C	109.5
N2—C7—C8	120.86 (15)	H15B—C15—H15C	109.5
N2—C7—H7A	119.6		
			
C1—N1—N2—C7	177.98 (15)	N1—C1—C6—C5	179.86 (16)
N2—N1—C1—C2	−179.34 (15)	C2—C1—C6—C5	−0.8 (2)
N2—N1—C1—C6	0.0 (2)	N1—N2—C7—C8	178.99 (14)
N1—C1—C2—C3	179.14 (16)	N2—C7—C8—C13	−178.30 (15)
C6—C1—C2—C3	−0.2 (2)	N2—C7—C8—C9	0.3 (2)
N1—C1—C2—N3	−3.1 (3)	C13—C8—C9—C10	0.7 (2)
C6—C1—C2—N3	177.54 (15)	C7—C8—C9—C10	−177.94 (16)
O2—N3—C2—C3	−9.4 (2)	C14—O5—C10—C9	−3.1 (2)
O1—N3—C2—C3	169.55 (15)	C14—O5—C10—C11	177.49 (14)
O2—N3—C2—C1	172.77 (16)	C8—C9—C10—O5	179.66 (15)
O1—N3—C2—C1	−8.3 (2)	C8—C9—C10—C11	−0.9 (2)
C1—C2—C3—C4	0.6 (2)	O5—C10—C11—O6	−0.2 (2)
N3—C2—C3—C4	−177.29 (15)	C9—C10—C11—O6	−179.65 (15)
C2—C3—C4—C5	0.0 (2)	O5—C10—C11—C12	179.98 (15)
C2—C3—C4—N4	179.57 (15)	C9—C10—C11—C12	0.5 (3)
O3—N4—C4—C3	−7.0 (2)	O6—C11—C12—C13	−179.69 (16)
O4—N4—C4—C3	173.20 (15)	C10—C11—C12—C13	0.1 (3)
O3—N4—C4—C5	172.60 (17)	C11—C12—C13—C8	−0.4 (3)
O4—N4—C4—C5	−7.2 (2)	C9—C8—C13—C12	−0.1 (2)
C3—C4—C5—C6	−1.0 (3)	C7—C8—C13—C12	178.60 (16)
N4—C4—C5—C6	179.47 (16)	C10—O5—C14—C15	−178.05 (14)
C4—C5—C6—C1	1.4 (3)		

### Hydrogen-bond geometry (Å, º)

**Table:**

*D*—H···*A*	*D*—H	H···*A*	*D*···*A*	*D*—H···*A*
O6—H1*O*6···O2^i^	0.84 (3)	2.21 (3)	2.986 (2)	155 (3)
O6—H1*O*6···O5	0.84 (3)	2.19 (3)	2.663 (3)	116 (2)
N1—H1*N*1···O1	0.86 (2)	2.007 (18)	2.641 (2)	130.0 (18)

Symmetry code: (i) *x*, *y*+1, *z*.
